# Supplementary material for: Real-World Outcomes of Subcutaneous PHESGO® in HER2-Positive Breast Cancer: Pathological Response, Sequencing, and Safety
Source: Curr Oncol. 2025 Nov 24;32(12):658. doi: 10.3390/curroncol32120658 (PMC12732124; doi:10.3390/curroncol32120658)
Supplement: Supplementary file 1 [file curroncol-32-00658-s001.zip › curroncol-3952458-supplementary.pdf]

File S1: STROBE Statement Checklist—Cohort Study (Retrospective)

Study: Real-World Outcomes of Subcutaneous PHESGO® in HER2-Positive Breast Cancer: Pathological Response, Sequencing, and Safety
Design: Single-center retrospective observational cohort
Setting: Nippon Medical School Tama-Nagayama Hospital, Tokyo, Japan (Jan 2024–Jul 2025)
Population: HER2-positive breast cancer patients receiving SC PHESGO® (neoadjuvant, adjuvant, or metastatic settings)
Ethics: IRB approved; informed consent via opt-out
Funding: None

Item	Recommendation	Location in Manuscript	Notes
1. Title/Abstract	Indicate study design	Title, Abstract	"Retrospective observational analysis" mentioned
2. Background	Explain rationale	Introduction	Unmet need for Asian real-world evidence
3. Objectives	State objectives/hypotheses	Abstract, end of Introduction	Primary: pCR in neoadjuvant; Secondary: sequencing, biomarkers, safety, metastatic efficacy
4. Study design	Present key elements early	Methods (2.1)	Retrospective cohort
5. Setting	Describe setting, locations, dates	Methods	Jan 2024–Jul 2025, single hospital
6. Participants	Eligibility, selection methods	Methods	HER2-positive, PHESGO® treated; subdivided by setting

7. Variables	Define outcomes, exposures, confounders	Methods	pCR definition, ER/PR, Ki-67, p53, LVEF thresholds
8. Data sources/measurement	Methods of assessment	Methods	EMR, pathology reports, ASCO/CAP HER2 guidelines
9. Bias	Describe efforts to address bias	Methods, Discussion	IPTW adjustment, note residual confounding
10. Study size	Explain sample size	Methods	Convenience sample, n=47; no power calc
11. Quantitative variables	Handling of quantitative data	Methods	Ki-67 continuous, age categorized (<70/≥70)
12. Statistical methods	Describe all methods	Methods	Fisher's, t/Mann–Whitney, logistic regression, FDR, IPTW
13. Participants	Report numbers at each stage	Results (3.1, Table 1)	Neoadjuvant=26, adjuvant=11, metastatic=10; suggest flow diagram
14. Descriptive data	Give characteristics	Results (Table 1)	Baseline demographics, biomarkers
15. Outcome data	Report outcome events	Results (Tables 2–3)	pCR, safety, metastatic efficacy

16. Main results	Provide estimates and precision	Results (3.2–3.3)	PHESGO-first superior pCR (65%; OR≈26.8)
17. Other analyses	Report subgroup/sensitivity analyses	Results (3.4)	IPTW, age-stratified, FDR adjustments
18. Key results	Summarize key outcomes	Discussion (opening)	Consistency with FeDeriCa, NeoSphere
19. Limitations	Discuss limitations	Discussion (mid–end)	Small sample, single center, short follow-up
20. Interpretation	Give overall interpretation	Discussion	Clinical implications and literature context
21. Generalisability	Discuss external validity	Discussion (end)	Relevance to Asian practice
22. Funding	Sources/role	Acknowledgements	“No external funding”; COI disclosed
